# Deep Learning-Based Prediction of Alzheimer’s Disease Using Microarray Gene Expression Data

**DOI:** 10.3390/biomedicines11123304

**Published:** 2023-12-13

**Authors:** Mahmoud M. Abdelwahab, Khamis A. Al-Karawi, Hatem E. Semary

**Affiliations:** 1 Department of Mathematics and Statistics, College of Science, Imam Mohammad Ibn Saud Islamic University, Riyadh 11564, Saudi Arabia; hesemary@imamu.edu.sa; 2 Department of Basic Sciences, Higher Institute of Administrative Sciences, Belbeis 44621, Egypt; 3 School of Science, Engineering and Environment, Salford University, Salford M5 4WT, UK; k.a.yousif@edu.salford.ac.uk; 4 College of Veterinary Medicine, Diyala University, Baquba 32001, Iraq; 5 Department of Statistics and Insurance, Faculty of Commerce, Zagazig University, Zagazig 44519, Egypt

**Keywords:** deep learning, convolutional neural networks (CNNs), Alzheimer’s, gene expression, microarray technique

## Abstract

Alzheimer’s disease is a genetically complex disorder, and microarray technology provides valuable insights into it. However, the high dimensionality of microarray datasets and small sample sizes pose challenges. Gene selection techniques have emerged as a promising solution to this challenge, potentially revolutionizing AD diagnosis. The study aims to investigate deep learning techniques, specifically neural networks, in predicting Alzheimer’s disease using microarray gene expression data. The goal is to develop a reliable predictive model for early detection and diagnosis, potentially improving patient care and intervention strategies. This study employed gene selection techniques, including Singular Value Decomposition (SVD) and Principal Component Analysis (PCA), to pinpoint pertinent genes within microarray datasets. Leveraging deep learning principles, we harnessed a Convolutional Neural Network (CNN) as our classifier for Alzheimer’s disease (AD) prediction. Our approach involved the utilization of a seven-layer CNN with diverse configurations to process the dataset. Empirical outcomes on the AD dataset underscored the effectiveness of the PCA–CNN model, yielding an accuracy of 96.60% and a loss of 0.3503. Likewise, the SVD–CNN model showcased remarkable accuracy, attaining 97.08% and a loss of 0.2466. These results accentuate the potential of our method for gene dimension reduction and classification accuracy enhancement by selecting a subset of pertinent *genes*. Integrating gene selection methodologies with deep learning architectures presents a promising framework for elevating AD prediction and promoting precision medicine in neurodegenerative disorders. Ongoing research endeavors aim to generalize this approach for diverse applications, explore alternative gene selection techniques, and investigate a variety of deep learning architectures.

## 1. Introduction

Alzheimer’s disease is a prevalent neurodegenerative disorder characterized by progressive memory and cognitive decline. It impacts many individuals worldwide, harming neurons associated with language and memory functions within the brain. The onset of symptoms typically occurs after the age of 65, and the prevalence of the disease increases substantially with advancing age. AD is one of the most common forms of dementia [1,2]. This chronic neurodegenerative condition manifests quietly and deteriorates gradually [3]. Alzheimer’s is a complex neurodegenerative disease with a significant genetic component. Understanding its development, progression, and pathophysiological mechanisms is crucial for advancements in research and therapeutic interventions. By 2015, the worldwide count of individuals impacted by AD had reached 47 million, driving associated costs beyond USD 818 billion. These numbers are projected to increase in the future [4]. AD is predicted to affect one in every eighty-five individuals by 2050 [5]. Traditionally, the diagnosis of AD has relied on brain magnetic resonance imaging (MRI) and neuropsychological testing [6,7]. Advancements in omics data have enabled the development of prediction techniques for AD, enabling machine learning models to diagnose the disease in its early stages, overcoming challenges in obtaining brain samples [7,8]. These methods offer the advantages of convenience and affordability to patients. Certain circumstances have shown that ML models can outperform clinicians in predicting AD [9]. Consequently, extensive research has explored the application of ML in AD diagnosis, utilizing medical data in various forms, including MRI scans [10]. AD, comprising 60–80% of all illnesses, presents a formidable challenge. By 2050, there is projected to be a significant rise in the number of affected individuals in the United States. The associated cost of dementia is estimated to reach USD 2 trillion globally, with affected individuals expected to increase from 5.4 million to 16 million. Despite these alarming statistics, the absence of a reliable pre-symptomatic detection method underscores the critical need for early intervention in disease progression [11]. Microarray technology is used to identify *genes* linked to AD, enabling biologists to evaluate gene expression levels, identify optimal treatments, and make accurate medical diagnoses [12,13,14]. Microarray data analysis presents challenges, including redundancy and overfitting due to the large number of *genes* and samples considered, requiring careful elimination and mitigation [15]. Scientists use the “gene selection approach” to identify relevant *genes* for disease classification, reducing computational costs and improving efficiency, particularly in AD [16]. PCA and SVD are unsupervised algorithms used for gene selection in gene expression microarray data analysis, providing insights into dataset structure and creating reduced-dimensional representations for classification tasks [17,18,19]. Microarray gene expression data is a promising avenue for the early detection of AD, compared to neuroimaging and EEG, which offer distinct advantages and considerations. Understanding their merits is crucial for advancing AD diagnostics. This method provides insights into disease molecular mechanisms and biological pathways, potentially identifying dysregulated *genes* and pathways, providing insight into disease etiology and progression. Moreover, microarray data can facilitate the development of robust biomarkers for AD. Microarray data may not accurately visualize brain abnormalities, but neuroimaging techniques like MRI and PET offer valuable insights into structural and functional changes in AD. They can help identify amyloid plaques, tau tangles, and atrophy patterns characteristic of the disease. Machine learning models applied to multimodal neuroimaging data have shown promising results in classifying different stages of AD. EEG, a non-invasive and cost-effective method for large-scale AD diagnostics, offers temporal information on brain activity, detects functional changes in early stages, and has potential for AD classification.

A multimodal approach combining microarray gene expression data with neuroimaging or EEG could enhance early detection, disease progression tracking, and personalized treatment strategies. This study uses a Deep Learning framework to identify Alzheimer’s using gene expression data. Convolutional Neural Networks are used to learn and discern unfamiliar class labels [20]. The research explores the potential of CNN architecture for enhanced predictive accuracy. The study focuses on gene selection methods and accurate categorization. The hypothesis is that deep learning models, particularly neural networks, can learn complex patterns in gene expression data, potentially distinguishing Alzheimer’s disease from healthy individuals and predicting its presence or absence based on microarray gene expression data.

The remaining research project components are as follows: Section 2 presents the related work. Section 3 provides a microarray technology. Section 4 outlines the experimental setup. Section 5 is the methodology, and Section 6 discusses the results. The study concludes with conclusions and suggestions for future investigations, detailed in Section 7.

## 2. Related Work

A study that used the Fisher method to identify disease-associated *genes* in a microarray dataset achieved a classification precision of 94.55% using only 44 *genes*. The method identifies 55% of AD cases with specific *genes* [3].

A study uses gene ontology and KEGG to identify AD-related terms and pathways, revealing potential disease predictors. Advancements in gene expression analysis and DNA microarrays provide robust data for disease diagnosis [21]. DNA microarrays offer valuable insights into the expression levels of numerous *genes* [22]. Gene expression levels signify the abundance of messenger ribonucleic acid (mRNA) molecules within the cell. Leveraging these levels, it becomes possible to detect diseases, identify optimal treatment options, and discover mutations in various biological processes [23]. In a notable example, researchers in [24] utilize gene expression biomarkers derived from blood samples to differentiate cases of Alzheimer’s disease. By employing XGBoost classification models, they successfully detect AD in a diverse aging population, incorporating relevant mental and elderly health disorders.

Nevertheless, there remains a necessity to augment the model’s sensitivity to establish a blood signature for AD that is more specific. In the context of [25], the researchers employ three distinct datasets—AddNeuroMed1 (ANM1), ANM2, and AD Neuroimaging Initiative (ADNI)—to distinguish individuals with AD from those who are cognitively normal (CN). The process incorporates gene selection (GS) methodologies to identify the most informative *genes.* These encompass variation auto encoders, transcription factors, hub *genes*, and convergent functional genomics (CFG). For the classification task, five distinct models are utilized: Support Vector Machine (SVM), Random Forest (RF), logistic regression (LR), L1-regularized LR (L1-LR), and Deep Neural Network (DNN). The achieved areas under the curve (AUC) values are 87.4%, 80.4%, and 65.7% for ANM1, ANM2, and ADNI, respectively. Furthermore, the authors examine the biological functions attributed to the blood *genes* associated with AD, juxtaposing them with the *genes* in the brain. The extraction yields 1291 brain *genes* from one gene expression dataset and 2021 blood *genes* from the other three datasets, with 140 *genes* shared between the two categories. In the context of [11], a study is conducted to identify the expressed genes within a blood dataset and examine the relationship between the *genes* expressed in both the blood and brain of patients with AD. The researchers successfully identified 789 differentially expressed *genes* shared by blood and brain samples. To perform gene selection (GS), the study employs the Least Absolute Shrinkage and Selection Operator (LASSO) regression. For classification tasks, logistic ridge regression (RR), Support Vector Machine (SVM), and Random Forest (RF) models are utilized. The study effectively distinguishes AD cases from control cases, yielding an accuracy rate of 78.1%. Within [26], the research endeavors to pinpoint potential diagnostic biomarkers for AD by examining gene expression data originating from multiple brain regions. In particular, gene expression data from six distinct brain regions are scrutinized to uncover indicative AD biomarkers. A *t*-test is used to identify *genes* with significant expression differences in AD and validate their biomarkers for clinical diagnosis. In [20], the researchers merge gene expression and DNA methylation datasets, resulting in a multi-omics dataset tailored for AD prediction. Li et al. use a deep neural network for accurate AD prediction using techniques like Principal Component Analysis and t-stochastic Nearest Neighbor. The study utilizes the GSE5281 dataset and employs a Wrapper of Genetic Algorithms and Support Vector Machine for feature selection. Six distinct classification methodologies, including Naive Bayes (NB), C4.5 (decision tree), K-Nearest Neighbor (KNN), Random Forest (RF), and Support Vector Machine with Gaussian and linear kernels, are implemented. The evaluation of classification models reveals varying accuracy rates: NB (81.4%), C4.5 (78.9%), KNN (87.0%), RF (87.0%), SVM with Gaussian kernel (85.7%), and SVM with linear kernel (91.9%) [27].

Balamurugan et al.’s research uses a K-Nearest Neighbor (KNN) classification algorithm to diagnose and classify AD and Mild Cognitive Impairment (MCI) using dimensionality reduction datasets. However, the KNN approach has limitations, especially when dealing with large amounts of information, requiring careful consideration of data quantity and relevance [28]. Karthik et al. use the Rhinoceros Search Technique and machine learning to compare AD patients’ and healthy individuals’ gene expression profiles. They discover 24 novel gene biomarkers using four supervised ML approaches: Support Vector Machines, Random Forest, Naive Bayes, and Multilayer Perceptron Neural networks. The RSA-MLP-NN model performs exceptionally well, distinguishing AD-associated *genes* from healthy ones [29]. Park et al. propose using deep learning techniques for predicting AD using large-scale gene expression (GE) and DNA methylation data in their research [20]. The “curse of dimensionality” in microarray data refers to the high number of features or variables compared to the limited sample size, causing issues like computational complexity, overfitting, and reduced generalizability.

Researchers use feature selection, dimensionality reduction, and regularization methods to improve accuracy and interpretability, leading to more biological insights [30]. The study highlights the challenges of modeling AD using a multi-omics dataset, focusing on differentially expressed *genes* and methylated positions to reduce feature count and handle large-sample data. The results were evaluated using the Area under the Curve (AUC) metric, yielding values of 0.79%, 0.75%, 0.77%, and 0.77% for the respective models. It is important to note that the study encountered limitations, including the highest feasible computing speed and other restrictions specified in the paper [31]. The time-consuming nature of microarray technology is primarily attributed to its intricate and multistep experimental process. This powerful tool for studying gene expression and genomic variations involves several meticulous stages, including sample preparation, RNA or DNA isolation, labeling, and hybridization on microarray chips. These preparatory steps demand precision and are inherently time-intensive. Following this, the data acquisition phase involves scanning and capturing data from numerous probes, which can be particularly time-consuming when handling multiple samples or replicates. Researchers then engage in data analysis, preprocessing, and identifying gene expression changes, constituting a time-intensive process involving quality control checks, robust design, result interpretation, validation, and resource availability. Despite the considerable effort involved, microarray experiments remain invaluable in genomic research.

## 3. Microarray Technology

**Figure 1:**
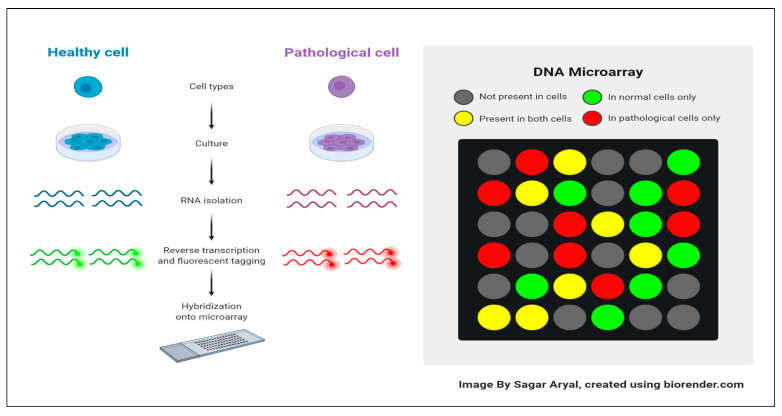
DNA microarray’s surface [41].

**Figure 2:**
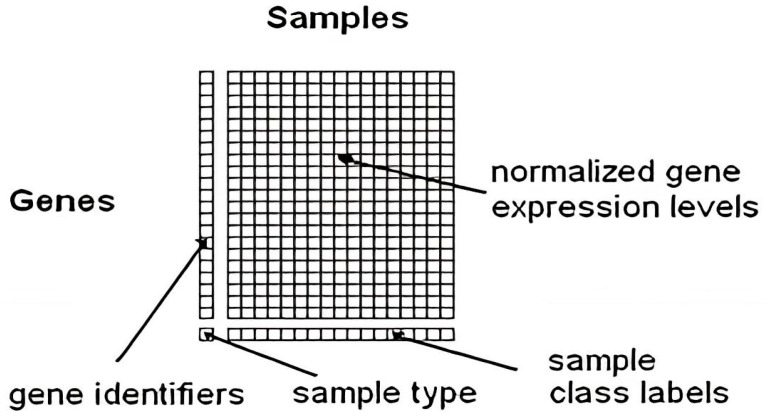
The gene expression data matrix [38].

Microarray technology is a widely used tool in molecular biology and genetics for studying gene expression on a large scale, enabling simultaneous analysis of thousands of *genes* in a single experiment [32]. Microarray technology immobilizes DNA or RNA probes onto a solid surface to complement specific *genes*, using a glass slide or microchip as the base [33]. The target *genes* are hybridized into a microarray using a fluorescent dye, with fluorescence signal intensity corresponding to the sample’s abundance of the target gene [34]. Microarray technology uses fluorescence signals to analyze gene expression profiles, enabling applications like biomarker discovery, disease identification, and drug target identification, revolutionizing fields like genomics, transcriptomics, and personalized medicine. However, it is important to note that newer sequencing-based technologies, such as RNA sequencing (RNA-seq), have primarily superseded microarray technology. RNA-seq provides more comprehensive and quantitative gene expression data and the ability to detect novel transcripts and splice variants. Nonetheless, microarray technology remains useful for specific applications and continues to be employed in many research laboratories [35]. Microarray technology is a method where nucleic acid fragments are attached to a chip and exposed to DNA or RNA, generating fluorescence. It is used in research and clinical studies to measure gene expression and detect specific DNA sequences. [36]. Microarray technology revolutionizes genomic analysis, enabling comprehensive studies in biology and biomedicine without sequencing and reducing the costs associated with large-scale studies [37]. Microarrays offer gene expression analysis and easy investigation of single nucleotide polymorphisms (SNPs), applicable in genome-wide association studies (GWAS) and applied to primary diseases, model organisms, and other organisms worldwide [34]. The expression level of each gene is stored in the form of an image (CEL File). Specialized software is subsequently employed to extract the data from these images [38]. Figure 1 illustrates the surfaces of a DNA microarray. Various microarray companies offer their proprietary software for analysis [39,40]. Moreover, Figure 2 Shows the gene expression data matrix [38].

## 4. Experiment Setup

The AD dataset, a combination of publicly available gene expression data from GSE63060 and GSE63061, contains 16,383 *genes* and 569 samples from individuals with Alzheimer’s, mild cognitive impairment, and healthy controls, offering a comprehensive resource for studying gene expression patterns.

### 4.1. Gene Selection

Microarray experiments enable the observation of gene expression differences across multiple conditions, resulting in significant data. However, the high dimensionality of microarray data poses challenges as most *genes* are unrelated to the classification process. To tackle this problem, researchers utilize gene selection techniques to pinpoint a subset of pertinent *genes*, simultaneously mitigating the complexity of the data [1]. These gene selection methods aim to identify a limited set of *genes* that yield optimal results [42], leading to improved accuracy of AD classifiers and reduced computational costs. In this investigation, gene selection approaches such as Principal Component Analysis (PCA) and Singular Value Decomposition (SVD) were employed to pinpoint *genes* directly affecting disease diagnosis. This study used gene selection techniques, including Singular Value Decomposition (SVD) and Principal Component Analysis (PCA), to pinpoint pertinent *genes* within microarray datasets. The utilization of Singular Value Decomposition (SVD) and Principal Component Analysis (PCA) for gene selection in microarray data analysis is well justified due to several compelling reasons. Microarray datasets have a high-dimensional feature space, leading to overfitting and computational challenges. SVD and PCA are effective in reducing dimensionality while preserving significant variation. They also reduce noise by focusing on critical components and enhancing signal-to-noise ratios. SVD and PCA also offer visualization, allowing researchers to project high-dimensional gene expression data into lower-dimensional spaces for data exploration and interpretation. The reduced feature space often includes linear combinations of *genes*, enhancing interpretability. These techniques are computationally efficient, making them suitable for large-scale data analysis. However, they may not fully capture the non-linear complexities of AD genetic data. Non-linear dimensionality reduction techniques, such as t-SNE, Isomap, or autoencoders, are needed to capture and represent non-linear relationships in data.

#### 4.1.1. Principal Component Analysis

Principal Component Analysis (PCA) is a widely embraced unsupervised technique for examining gene expression data, providing valuable insights into the underlying data structure. Among its applications, PCA stands out as one of the most potent gene selection methods available [43]. Its primary aim is to transform high-dimensional data into a new, lower-dimensional subset while retaining crucial details from the original dataset. Before deeper investigations, a substantial amount of valuable gene information is distilled from extensive datasets using PCA testing [43]. Principal Component Analysis (PCA) is an unsupervised method used in gene selection to prevent overfitting, enhance accuracy, maintain model simplicity, and improve classification accuracy. It helps identify influential *genes* contributing to data variation, providing a focused subset for further analysis. PCA is commonly used in bioinformatics and genomics to reduce high-dimensional gene expression data while retaining variance. An unsupervised method based on eigenvector analysis is recommended. A dataset containing m-dimensional data, PCA transforms the m-dimensional data into a k-dimensional subspace (where k < m). The following steps outline the PCA process [44]. Assuming we have an input matrix X consisting of m-dimensional n-vectors, let us calculate the mean data ($\bar{X}$) for each dimension using Equation (1):

(1) $$\bar{X}=\frac{1}{n}\sum_{i=1}^{n}X_{i}$$

where:

*N* is the number of samples, and *X_i_* represents the value of item *i.*

In the next step, to compute the covariance matrix (*Cx*), you can use the following Equation (2) [45]:

(2) $$C_{x}=\frac{1}{n-1}\sum_{i=1}^{n}(X_{i}-\bar{X}){\left(X_{i}-\bar{X}\right)}^{T}$$

where:

*n* is the number of samples, *X* is the original data matrix and $\bar{X}$ is the mean data for each dimension. The third step is to determine the eigenvalues (*λ_m_*) and eigenvectors (*Vm*) of the correlation matrix; you can use Equation (3):

(3) $$C_{x}V_{m}=\lambda_{m}V_{m}$$

In Step 5, the eigenvalues are organized in descending order. Correspondingly, a set of eigenvectors, known as the principal components (PCs), aligns with the sorted eigenvalues. Ultimately, the eigenvalues play a critical role in defining the strategy for reducing the dimensionality of the principal components (PCs).

The equations for performing Principal Component Analysis (PCA) involve linear algebra operations to compute the principal components and select *genes* based on their contributions to these components. Given a gene expression matrix *X* of size *n × p*, where *n* is the number of samples and *p* is the number of *genes*, the goal is to identify the most important *genes* associated with the principal component.

#### 4.1.2. Singular Value Decomposition (SVD)

Singular Value Decomposition (SVD) of the matrix *X* involves breaking down the matrix X into a series of matrices, where *X* represents the gene expression data matrix with dimensions *d × n* [45].

(4) $$X=U\sum V^{T}=\sum_{i=1}^{r}\lambda_{i}u_{i}v_{i}^{T}$$

In the context where “*r*” represents the rank of matrix *X*, we have the following matrices:

*U* = [*u*_1_, *u*_2_… *u_r_*], a matrix with dimensions *d × r* featuring orthonormal columns.

V = [*v*_1_, *v*_2_… *v_r_*], a matrix with dimensions *N × r* comprising orthonormal columns.

Σ, a matrix with dimensions *r × r* wherein the elements

*λ*_1_ (*λ*_1_ > 0, 1 ≤ *I ≤ r, λ*_1_ ≥ *λ*_2_ *≥…*≥ *λ_r_*) are situated along the diagonal of the matrix.

#### 4.1.3. Feature Modalities

Feature modalities are various features used in data analysis, machine learning, and statistical modeling, particularly in complex applications like image analysis or natural language processing. Consider matrix *C_i_* as the rows of the matrix *ΣV^T^*; by applying Equation (4), we obtain:

(5) $$\begin{array}{l} X-\sum_{i=1}^{t}\left(u_{i}\right){\left(\lambda_{i}v_{i}\right)}^{T}\\ =[u_{1}\sqrt{\lambda_{1}},u_{2}\sqrt{\lambda_{2}},K,u_{r}\sqrt{\lambda_{r}]}[\sqrt{\lambda_{1}v_{1}},\sqrt{\lambda_{2}v_{2}},K\cdot \sqrt{{\lambda_{r}v_{r}]}^{T}}\\ =RC^{T} \end{array}$$

**Figure 3:**
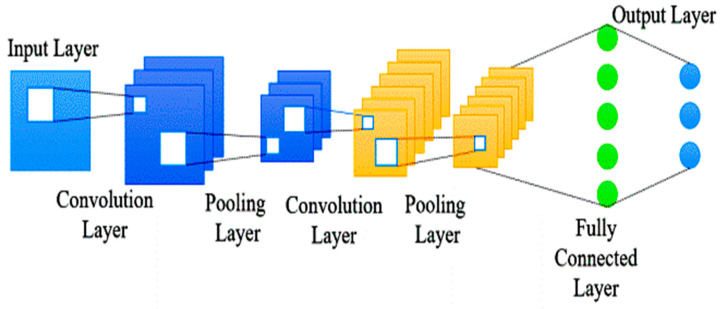
Convolutional neural networks [31].

Characteristics associated with matrix *X* are represented as orthogonal vectors *C_i_*. The expression of the *j*th gene varies among the examined samples, as outlined in Equation (3). Within this equation, the coefficients of this combination align with the respective entries in matrix *X*, allowing for a precise representation as a linear combination of the characteristic modes present in row *X_j_* of matrix *X*. Typically, only a subset of these distinct modes is required to reconstruct the gene expression pattern effectively. Figure 3 illustrates utilizing an incomplete SVD expression for this purpose.

(6) $$X_{j}=\sum_{i=1}^{l}U_{ji}C_{i}\;l\leq r$$

Equation (6) offers valuable insights into the examination of singular values and demonstrates how the dimension of matrix *X* can be diminished by reducing the dimension of the characteristic mode matrix. Additionally, the initial pattern of gene expression data can be described utilizing *C_i_* [45].

### 4.2. Deep Learning for Alzheimer’s

Deep learning is a subset of artificial intelligence that uses algorithms to create abstract representations by examining hidden patterns within datasets. It is used in automatic detection systems, disease classification, and instant medical diagnosis, offering improved outcomes and a deeper understanding of complex networks [46]. Convolutional Neural Networks (CNNs) are particularly common among the various architectures used in deep learning. CNNs have proven highly effective in classifying AD based on gene expression information.

#### Convolutional Neural Networks (CNN)

The Convolutional Neural Network is a deep learning technique inspired by the information-processing function of the brain. In this study, the use of a multilayer CNN is proposed to analyze gene expression patterns obtained from microarrays. The CNN architecture is recommended due to its ability to handle large volumes of data, leading to improved accuracy in data categorization. One key advantage of CNN is its capability to effectively merge closely related datasets, thereby enhancing the performance of classification tasks. This is especially valuable when distinguishing subtle AD characteristics from other diseases [31]. A seven-layer Convolutional Neural Network (CNN) with diverse configurations is a versatile deep learning model for computer vision tasks like image classification, object detection, and facial recognition. Its adaptability allows it to be fine-tuned to specific datasets and objectives, delivering optimal performance and robust results in complex or niche tasks. CNNs are effective in various fields, including gene expression analysis, image recognition, natural language processing, and speech recognition. They extract meaningful features from complex data, handle high-dimensional inputs, and learn hierarchical representations. CNNs integrate selection and classification processes, handling significant inputs efficiently while maintaining high computational efficiency. They are robust against minor data alterations and can take real-time data and cost-effective technology, making them a promising choice for various applications [31]. The 2D CNN model, as depicted in Figure 3, efficiently processes and extracts features from two-dimensional data like images, making it a versatile tool for various applications. This research used gene expression as input and incorporated convolutional layers, densely connected layers, and a flattening layer [47]. The CNN architecture effectively handles gene expression data, extracting essential features through convolution layers, performing deep learning and classification tasks through dense layers, and converting the output into suitable formats.

## 5. Methodology

**Figure 4:**
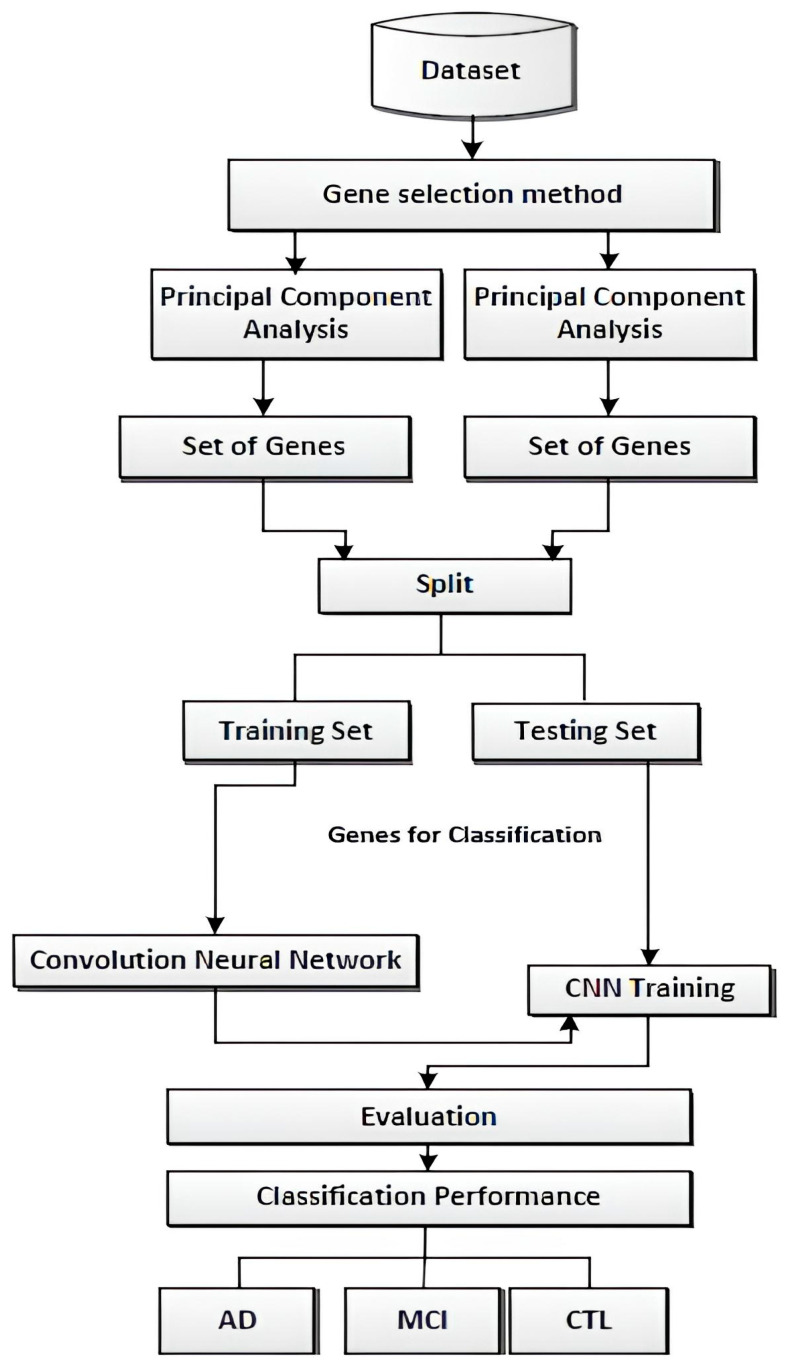
Proposed method [52].

The proposed methodology involves the initial step of importing raw microarray data for AD analysis. Subsequently, the data undergo normalization using the Min–Max method, a widely used data preprocessing technique. Min–Max normalization scales and transforms features within a specified range, preserving the original data relationships while ensuring values fall within the designated range. This technique is precious for machine learning algorithms as it enhances data interpretability. However, the choice of normalization method, whether Min–Max, Z-score, or robust normalization, depends on the nature of the data and specific requirements. Following normalization, essential procedures such as gene selection techniques are implemented to identify relevant *genes* and reduce data dimensionality. This study employs a Convolutional Neural Network (CNN) classifier to analyze complex patterns in gene expression data. The CNN leverages the hierarchical structure of the data and convolutional layers to extract intricate features, making it a robust choice for discerning patterns in genomic information. Figure 4 shows the proposed method.

### 5.1. Preprocessing

Pre-processing is a crucial step before conducting any analysis, particularly in the context of gene expression data. The primary objective is reducing variation in expression measurements, ensuring the dataset is standardized and suitable for subsequent analyses. Standardization procedures, such as Z-score normalization or Min–Max scaling, help bring all data points onto a common scale, making comparing and analyzing them more easy. This normalization process is essential for mitigating biases and inconsistencies in the raw data, providing a more robust foundation for downstream analyses. Overall, pre-processing serves as a critical preparatory phase, enhancing the quality and reliability of the data for further investigation [48]. Normalization is a crucial step in data preprocessing, especially when dealing with datasets that display substantial variations in the range of values, particularly after encoding nominal values into real numbers (1 and 2). The absence of normalization can lead to specific attributes with broader numeric ranges dominating over those with smaller ranges, introducing potential biases into the analysis. Beyond addressing these biases, normalization also plays a role in expediting the execution of algorithms by preventing the utilization of wide-ranging numbers. This ensures a more balanced and efficient analysis, contributing to the overall robustness of the subsequent data-driven processes [49].

In this context, data scaling is executed to confine values within the interval of 0 to 1, adhering to Equation (1). In this equation, *x* denotes the original value of the attribute, *x* normalized represents the scaled value, min *a* is the minimum value of attribute *a*, and max *a* is the maximum value of the attribute. Applying this scaling operation transforms each attribute’s values to a standardized range, facilitating uniformity and comparability across different features within the dataset. This normalization process is instrumental in ensuring that the subsequent analyses are not influenced by the original scale of the attributes, contributing to a more robust and reliable exploration of the data.

(7) $$X_{Normalized}=(\frac{x-\min_{a}}{\max-\min_{a}})$$

### 5.2. Gene Selection

Gene selection strategies play a pivotal role in data analysis by mitigating computational space’s dimensionality, focusing on pertinent *genes* within extensive datasets. Utilizing methods such as PCA (Principal Component Analysis) and SVD (Singular Value Decomposition), these strategies effectively identify and retain *genes*, significantly influencing classification outcomes. This approach optimizes categorization performance, enhancing result accuracy and interpretability. Gene selection becomes imperative in the realm of genomic data analysis, particularly in high-dimensional datasets like microarrays or RNA sequencing. Its impact notably reduces data dimensionality while preserving informative attributes, thereby improving downstream analyses. This process unveils biological insights, identifies biomarkers, and elucidates molecular mechanisms within complex diseases. The outcomes contribute to precise decision-making in medicine, biotechnology, and molecular biology [50].

The classification stage employs multiple layers, with the convolutional layer architecture being crucial for efficiently handling large volumes of multidimensional data, such as gene expression data [31]. This research introduces an innovative technique that employs a two-dimensional convolutional layer. This layer incorporates 65 kernels with a size of 4 and utilizes Rectified Linear Units (ReLU) as the activation function. ReLU is mathematically represented using the formula in Equation (8). The function *g*(*X*) = 0 for *X* < 0 indicates that *g*(*X*) equals zero when the input value *X* is less than zero. In other words, for all values of *X* that are negative or less than zero, the function *g*(*X*) is set to zero. This can be written as:

*g*(*X*) = 0 *for X* < 0 (8)

This study used CNN architecture with six layers for *X* values greater than or equal to 0, with a 100-norm input layer and 1,389,043 trainable parameters. The Softmax activation function enhanced network performance in the final layer. The Softmax function for the jth element (*C_j_*) can be defined as follows:

*SoftMax* (*C_j_*) = *e*^(*C_j_*)/*Σ*(*k* = 1 *to k*)* e*^(*C_k_*) (9)

In this equation, *e^*(*C_j_*) represents the exponential of the *j*th element *C_j_*, and *Σ* (k = 1 to *k*)* e*^(*C_k_*) is the sum of the exponentials of all elements in the set {*C*_1_, *C*^2^*… C_k_*}. The ADAM optimizer assesses the loss of training and testing data by utilizing a predefined objective function, categorical cross-entropy [51]. The ADAM optimizer calculates an individual learning rate for each parameter. In the system’s construction, the training data comprise 82% of the dataset, while the testing data account for 23%.

### 5.3. Evaluation Measures

Accuracy is a crucial performance metric in AD categorization, indicating the ratio of correctly predicted observations to total observations. Choosing between groups may require data preprocessing, feature extraction, and exploratory analysis, often requiring advanced techniques. The study uses Equations (9) and (10) to calculate accuracy and loss, respectively, to assess the effectiveness of a proposed methodology for accurately categorizing AD. Accuracy is calculated using the formula:

*Accuracy* = (*TP* + *TN*)/(*TP* + *TN* + *FP* + *FN*) × 100 (10)

The loss function is a crucial tool in the proposed method, indicating the error score and its effectiveness in dealing with categorical outcomes beyond binary ranges. The loss is computed as: −*Xi′log*2(*Xi*/*Ni*), where *N* represents the count of samples in the *i*th instance. *Xi′* corresponds to the true class label, and *Xi′* stands for a projected label. These metrics collectively contribute to comprehensively evaluating and improving the approach’s performance.

## 6. Results and Discussion

AD presents a complex landscape characterized by variations in prevalence, clinical presentation, and risk factors across diverse demographic groups, including distinctions related to age, gender, ethnicity, and socioeconomic status. To ensure the practicality and generalizability of machine learning models for AD detection, it is imperative to conduct thorough validation and testing across a wide array of datasets representing these distinct population segments. In this study, we harnessed the power of Principal Component Analysis (PCA) and Singular Value Decomposition (SVD) as robust gene selection methods to enhance the efficiency and efficacy of our classification algorithm. Our primary objectives were first to identify the optimal number of *genes* required for accurate disease identification and second to execute the classification of gene expression data. To accomplish this, we initiated the process by reading and normalizing the gene expression data through the Min–Max approach, ensuring consistency and reliability in our analyses. To align the gene count with the available samples in our dataset, we strategically employed gene selection techniques, relying on the principles of PCA and SVD. These techniques were carefully applied to reduce the number of *genes*, ultimately curating a more refined and informative subset. The crucial advantage of this approach lies in its ability to eliminate irrelevant *genes*, which are often found within the initial dataset but contribute minimally to the predictive power of class labels. Table 1 shows a succinct overview of the selected data, presenting a detailed account of the *genes* identified through the proposed gene selection methods. By delineating these selections, we emphasize this process’s significance in enhancing our classification algorithm’s overall performance. It is worth noting that, in our pursuit of precision and efficiency; we have effectively trimmed away superfluous *genes*, a pivotal step towards a more accurate and streamlined approach to AD detection. This curation process stands as a testament to our commitment to advancing the field of neurodegenerative disorder research.

Our approach, combining PCA and CNN, significantly improves classification accuracy on the AD dataset, achieving an impressive 96.60% accuracy and demonstrating its effectiveness in different sample classes. Table 2 shows high accuracy and consistent performance over time, with the Singular Value Decomposition (SVD)-based gene selection method achieving a 97.08% accuracy rate when integrated with a CNN model, highlighting their effectiveness in gene selection. PCA and SVD techniques effectively identify and retain relevant gene features for adequate classification. Their utility extends beyond gene selection, advancing diagnostics, personalized medicine, and understanding complex biological systems. Their ability to improve machine learning models and genomics applications demonstrates their potential in gene selection tools. The use of microarray gene expression data for Alzheimer’s diagnostics, alongside neuroimaging and EEG, is a complex technique that faces competition from other diagnostic modalities. Each method offers distinct advantages and considerations, and understanding their relative merits is crucial for advancing AD diagnostics. Microarray gene expression data analysis has emerged as a promising avenue for early AD detection. This method offers insights into the underlying molecular mechanisms and biological pathways associated with the disease. It can potentially identify dysregulated *genes* and pathways, shedding light on disease etiology and progression.

Moreover, microarray data can facilitate the development of robust biomarkers for AD. Microarray data may not accurately visualize brain abnormalities, but neuroimaging techniques like MRI and PET can help identify structural and functional changes in AD. Detecting AD early is crucial for timely intervention, and MRI measures brain volume decline in the mesial temporal cortex. The author introduces a heuristic early feature fusion framework that combines PET and MRI images, trains a modified Resnet18 deep learning architecture, and extracts informative features for effective binary AD classification, enhancing the performance of AD diagnostic methods. The experimental results demonstrate the model’s effectiveness, achieving a classification accuracy of 73.90% when tested on the ADNI database. Machine learning models applied to multimodal neuroimaging data have shown promising results in classifying different stages of AD. However, these approaches may be costlier and less accessible for routine screening than gene expression data analysis.

EEG has the advantage of being non-invasive and cost-effective, making it suitable for large-scale AD diagnostics. It provides temporal information on brain activity and can be valuable in detecting functional changes in early AD stages. Deep learning models applied to EEG data have shown potential for AD classification.

Nevertheless, EEG may have limitations in capturing specific structural brain alterations, which can be more effectively visualized through neuroimaging. It is worth emphasizing that a multimodal approach, integrating microarray gene expression data with neuroimaging or EEG, may offer a comprehensive perspective on AD diagnostics. Such an approach can potentially leverage the strengths of each modality to improve early detection, track disease progression, and personalize treatment strategies.

Further research and collaboration across these domains are essential to refine diagnostic methods and achieve more accurate, timely, and accessible AD diagnoses. A validation set was employed as part of our model evaluation process. The validation set was critical in assessing how well our model generalizes to unseen data, ensuring that our results are reliable and do not overfit the training data. In our evaluation, we closely monitored the model’s performance on the validation data and compared it to its performance on the training data.

In the context of our study, addressing overfitting was a critical aspect of ensuring the robustness and generalizability of our model. Overfitting occurs when a model learns the underlying patterns in the training data and captures noise and fluctuations, leading to poor performance on unseen data. Several strategic steps were implemented to mitigate overfitting in our approach. Data augmentation techniques were employed to artificially expand the training dataset, introducing variations such as rotations, flips, and zooms. This ensured the model learned more generalized features and reduced its susceptibility to memorizing noise in the training data. Dropout randomly deactivated a proportion of neurons during training, preventing the model from relying too heavily on specific nodes and enhancing its adaptability to diverse patterns. Cross-validation played a pivotal role in assessing the model’s performance across different subsets of the data to gauge its generalization capability. Hyperparameter tuning was conducted meticulously, optimizing the network’s complexity to balance expressiveness and prevent overfitting. Early stopping mechanisms were implemented based on the model’s performance on a validation set, preventing it from further learning the training data when optimal performance was reached. These steps collectively contributed to building a resilient model that is robust against overfitting and capable of delivering consistent performance on new and unseen data.

**Figure 5:**
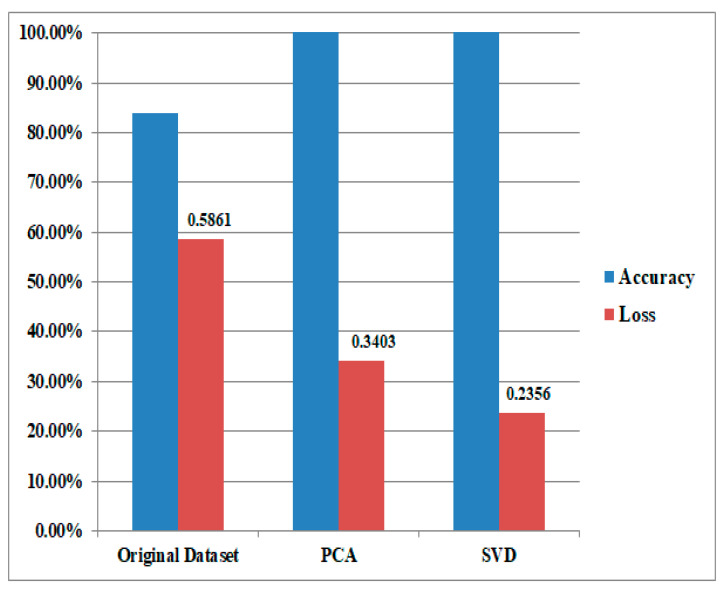
The power of the Principal Component Analysis (PCA) and Singular Value Decomposition (SVD) as robust gene selection methods to enhance the efficiency and efficacy of the classification algorithm.

In validating our model, a crucial step involved comparing its performance on the validation data to that on the training data using a technique known as cross-validation. This method partitions the dataset into multiple subsets, using one portion for training and the remainder for iterative validation. The model’s effectiveness was assessed by evaluating various metrics such as accuracy, precision, recall, and F1 score on both the training and validation sets. This approach provided valuable insights into how well the model generalized to new, unseen examples, offering a robust measure of its performance beyond the initial training data. Consistency in performance across these subsets demonstrated the model’s ability to handle diverse instances, while any significant deviations raised flags about potential overfitting. This thorough validation ensured the model’s reliability and applicability in real-world scenarios. Figure 5 shows the power of the Principal Component Analysis (PCA) and Singular Value Decomposition (SVD) as robust gene selection methods to enhance the efficiency and efficacy of the classification algorithm.

## 7. Conclusions

In conclusion, our research significantly advances AD prediction by utilizing deep learning techniques applied to microarray gene expression data. The development of accurate, early diagnostic methods for AD is paramount for timely intervention and personalized treatment. Our study demonstrates the potential of deep learning models for effectively classifying AD and contributes to a broader understanding of the role of microarray gene expression data in this context. We have introduced a novel approach that leverages the power of deep neural networks to extract informative features from complex gene expression datasets, facilitating accurate binary classification of AD. Complex multiclass microarray sample categorization was solved using a CNN model in this study. Due to the limitations of high-dimensional data, this work used PCA and Singular Value Decomposition for gene selection. These methods reduce data dimensionality, crucial for gene expression data processing. The proposed method was evaluated using accuracy and loss measures. To evaluate the model, the classification cross-entropy loss function frequently used for non-binary categorization problems was applied. The CNN model was fine-tuned using ADAM to converge efficiently to the ideal solution. PCA and SVD gene selection methods are used in this work to address data dimensionality issues. Accuracy and loss serve as indicators of the approach’s effectiveness. Classification cross-entropy, a loss function used in non-binary categorization, is used.

This work uses PCA and SVD gene selection methods to address data dimensionality issues. Accuracy and loss are indicators of the approach’s effectiveness. Classification cross-entropy is used in non-binary categorization. The model is optimized using ADAM. The method mitigates high-dimensional data problems, improves classification accuracy by creating a relevant subset, reduces processing time, and enhances classification efficacy. Several strategic steps were implemented to mitigate overfitting in our approach. Data augmentation techniques were employed to artificially expand the training dataset, introducing variations such as rotations, flips, and zooms. This ensured the model learned more generalized features and reduced its susceptibility to memorizing noise in the training data.

The proposed methodology, which incorporates Principal Component Analysis (PCA) and a Convolutional Neural Network (CNN) model, significantly improves classification accuracy in the Alzheimer’s dataset. The method achieves an accuracy rate of 95.70% and 96.09% for PCA and SVD, respectively. However, further real-world implications and integration into diagnostic workflows are needed. The study contributes to Alzheimer’s diagnosis by showcasing deep learning’s potential for early and accurate detection.

## Figures and Tables

**Table 1:** A concise overview of the chosen dataset.

Technique	Samples	Genes	Genes That Have Been Chosen
PCA Technique			530
SVD Technique	566	16,382	470

**Table 2:** The mean accuracy and loss values.

Technique	CNN	
	Performance	Loss
Initial Dataset	83.832	0.5861
PCA Technique	95.70	0.3403
SVD Technique	96.09	0.2356

## Data Availability

Data are contained within the article.
